# Evaluation of the Strain *Bacillus amyloliquefaciens* YP6 in Phoxim Degradation via Transcriptomic Data and Product Analysis

**DOI:** 10.3390/molecules24213997

**Published:** 2019-11-05

**Authors:** Di Meng, Liyuan Zhang, Jie Meng, Qiaopeng Tian, Lixin Zhai, Zhikui Hao, Zhengbing Guan, Yujie Cai, Xiangru Liao

**Affiliations:** 1 The Key Laboratory of Industrial Biotechnology, Ministry of Education, School of Biotechnology, Jiangnan University, Wuxi 214122, China; mengdi030321@126.com (D.M.); zly15906198696@163.com (L.Z.); hnyy816@163.com (Q.T.); E-jamie@outlook.com (L.Z.); 7160201008@vip.jiangnan.edu.cn (Z.G.); gsyyx2017@163.com (Y.C.); 2 Guangdong Key Laboratory for Innovative Development and Utilization of Forest Plant Germplasm, College of Forest and Landscape Architecture, South China Agricultural University, Guangzhou 510642, China; mengjiej@yeah.net; 3 Institute of Applied Biotechnology, Taizhou Vocational & Technical College, Taizhou 318000, China; 15852833873@163.com

**Keywords:** organophosphorus pesticides, *Bacillus amyloliquefaciens* YP6, response surface methodology, phoxim, transcriptome analysis, degradation pathway

## Abstract

Phoxim, a type of organophosphorus pesticide (OP), is widely used in both agriculture and fisheries. The persistence of phoxim has caused serious environmental pollution problems. In this study, *Bacillus amyloliquefaciens* YP6 (YP6), which is capable of promoting plant growth and degrading broad-spectrum OPs, was used to study phoxim degradation. Different culture media were applied to evaluate the growth and phoxim degradation of YP6. YP6 can grow rapidly and degrade phoxim efficiently in Luria–Bertani broth (LB broth) medium. Furthermore, it can also utilize phoxim as the sole phosphorus source in a mineral salt medium. Response surface methodology was performed to optimize the degradation conditions of phoxim by YP6 in LB broth medium. The optimum biodegradation conditions were 40 °C, pH 7.20, and an inoculum size of 4.17% (*v*/*v*). The phoxim metabolites, *O*,*O*-diethylthiophosphoric ester, phoxom, and α-cyanobenzylideneaminooxy phosphonic acid, were confirmed by liquid chromatography–mass spectrometry. Meanwhile, transcriptome analysis and qRT-PCR were performed to give insight into the phoxim-stress response at the transcriptome level. The hydrolase-, oxidase-, and NADPH-cytochrome P450 reductase-encoding genes were significantly upregulated for phoxim hydrolysis, sulfoxidation, and o-dealkylation. Furthermore, the phoxim biodegradation pathways by YP6 were proposed, for the first time, based on transcriptomic data and product analysis.

## 1. Introduction

Phoxim (*O*,*O*-diethyl *O*-(alpha-cyanobenzylideneamino) phosphorothioate), a type of organophosphorus pesticide (OP), is widely used in both agriculture and fisheries to control a variety of unwanted insects and underground pests via dipping, spraying, or pour-on applications [1]. Phoxim mainly inhibits the activity of acetylcholinesterase, leading to the accumulation of acetylcholine in the postsynaptic membrane and causing neurological disorders and biological poisoning [2]. However, as a result of its continuous and extensive utilization, this pesticide is frequently detected in food, soil, agricultural effluent, and river water [3,4,5], causing serious concerns over the environment and food safety.

Currently, physical degradation, chemical degradation, and biodegradation are, essentially, the methods to eliminate OP pollution from environments [6]. Biodegradation is the main method because it is low-cost, effective, and environmentally friendly [7,8]. To date, many microorganisms that efficiently degrade phoxim have been successfully isolated [9,10,11,12,13,14,15]. Among these phoxim-degrading bacteria, the genus *Bacillus* has a sporulation capacity that enables them to survive in adverse environmental conditions [16,17]. Furthermore, some *Bacillus* species also have other advantages, such as promoting plant growth [14] and producing antibiotics/metabolites against pathogenic microorganisms [17,18]. Multifunctional *Bacillus* species have become a hot research topic in various fields. However, most studies have focused on their biocontrol and plant growth-promoting ability [14,17,18]. Some studies have investigated the degradation of OPs by *Bacillus* members [19,20,21], but few have focused on the factors affecting OP degradation and the degradation mechanism. Hence, exploring the factors affecting OP degradation and the degradation mechanism can provide a theoretical basis for environmental remediation.

*Bacillus amyloliquefaciens* YP6 was isolated from the rhizosphere of *Lolium perenne* L. in a rock phosphorus mine in our previous research. Our preliminary results indicated that strain YP6 possessed plant growth-promoting ability [22] and could efficiently degrade a wide range of OPs, including phoxim [23,24]. In addition, we also sequenced and reported the complete genome of strain YP6 [24]. The aims of this paper were to (1) study the growth and phoxim degradation of strain YP6 in different media; (2) obtain the optimal conditions for the biodegradation of phoxim, including initial inoculum, incubation temperature, and pH; (3) analyze the metabolites of phoxim; (4) give insights into the phoxim-stress response at transcriptome level via RNA-seq technology and qRT-PCR; and (5) speculate the proposed pathways for the degradation of phoxim.

## 2. Results and Discussion

### 2.1. The Growth and Phoxim Degradation of Strain YP6 in Different Culture Media

LB broth (containing 50 mg L^−1^ of phoxim), M-1 (Table S1), M-2 (Table S2), and M-3 (Table S3) agar media (each containing 50 mg L^−1^ of phoxim) were utilized to evaluate the growth status of strain YP6. As shown in Table S4, strain YP6 could grow on LB broth (containing an initial phoxim concentration of 50 mg L^−1^) and M-2 agar medium, but not grow on M-1 and M-3 agar media, indicating that YP6 can use phoxim as a sole phosphorus source.

**Figure 1:**
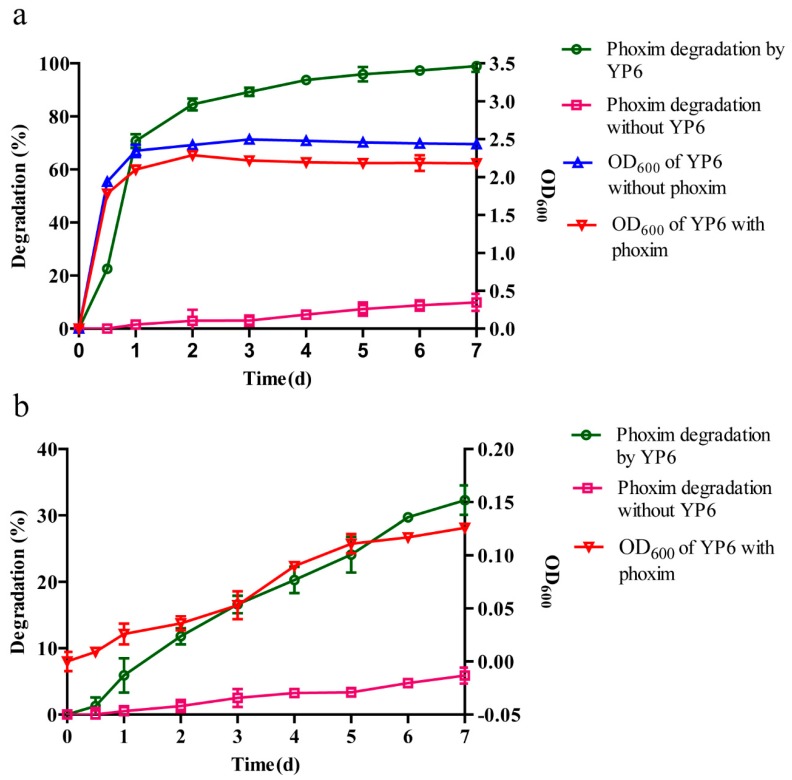
The growth and phoxim degradation of strain YP6 in different culture media. (**a**) The growth and phoxim degradation of strain YP6 in an LB broth medium (containing an initial concentration of 50 mg L^−1^ phoxim); (**b**) the growth and phoxim degradation of strain YP6 in an M-2 medium (containing an initial concentration of 50 mg L^−1^ phoxim). The bars represent the standard errors of assays performed in triplicate.

To study further the growth and phoxim degradation of strain YP6 in LB broth (containing an initial phoxim concentration of 50 mg L^−1^) and M-2 liquid medium, samples were taken at different times for analysis. In LB broth (Figure 1a), strain YP6 was able to grow both with and without the phoxim supplement, but the presence of the pesticide decreased the biomass yield, indicating that phoxim affects the growth of strain YP6. In addition, strain YP6 efficiently degraded phoxim. In the M-2 medium (Figure 1b), strain YP6 grew slowly with the reduction of phoxim. Comparing the growth and phoxim degradation of strain YP6 in the two media, strain YP6 grew faster and degraded more phoxim in LB broth. These results suggested that culture conditions play an important role on the growth and phoxim degradation of strain YP6. As most of the current studies have focused on the performance of OP degradation by microbes in a mineral salt medium (MSM) [15,25,26], and very few have concentrated on OP degradation in an LB broth medium, therefore an LB broth medium (containing an initial phoxim concentration of 50 mg L^−1^) was chosen for further study.

### 2.2. Optimal Condition for the Degradation of Phoxim by Strain YP6

In the present study, response surface methodology (RSM) was used to optimize the three variables (incubation temperature, pH, and initial inoculum size) affecting the degradation of phoxim. Table 1 shows the phoxim degradation corresponding to the combined effect of the three factors in the specified ranges. The results of quadratic model fitting in the form of analysis of variance (ANOVA) shown in Table 2 indicates that the response was significant (*p* < 0.0001), whereas the *p*-value for the lack of fit was not significant (*p*-value of 0.0808), demonstrating that the degree of fitting was good. The determination coefficient (R^2^ = 0.9973) indicated that 99.73% of responses were covered by the model, demonstrating that predicted values of the model were in good agreement with the experimental values [26,27]. Therefore, it was concluded that the model was satisfactory and could be used for the optimal conditions of phoxim degradation. Based on the model, the optimal phoxim degradation condition is obtained at a temperature of 40 °C, pH 7.20, and inoculum size of 4.17% (*v*/*v*). To further confirm the validity of the model predictions, three repeated experiments were performed at optimal factor levels. Under the optimal conditions, the predicted phoxim degradation was 96.44% and the actual recorded degradation was 96.12% ± 0.19% within 48 hours of cultivation. The predicted value was very close to the measured value, indicating high model validity in the RSM experiment.

Whereas in the review of Cycoń et al. [28], the optimal conditions of most OP-degrading microorganisms were 25 to 37 °C and pH 6.5–8.0, in the present studies, the optimum pH 7.0 was close to that of most microorganisms, but the optimum temperature 40 °C was higher. It is possible that some key enzyme(s) involved in phoxim degradation have their optimum enzymatic activity at a high temperature. Although the optimum degradation temperature was 40 °C, the OD_600_ of strain YP6 grown at 40 °C was lower than at 30 °C after 12 h (Figure S1). Therefore, 30 °C was chosen for sample preparation for transcriptome analysis and qRT-PCR validation.

### 2.3. Identification of Metabolites of Phoxim by Strain YP6

**Figure 2:**
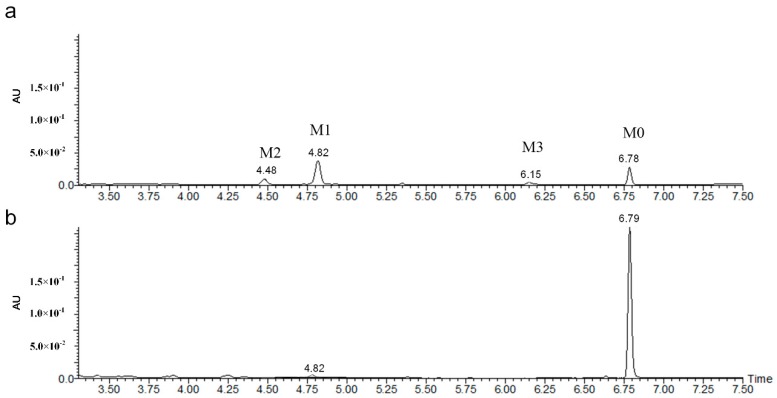
HPLC analysis of metabolic products of phoxim degraded by strain YP6. (**a**) Extract obtained from a culture of YP6 grown on LB broth plus 50 mg L^−1^ phoxim for 48 h; (**b**) extract obtained from an equivalent medium incubated without YP6 for 48 h.

**Figure 3:**
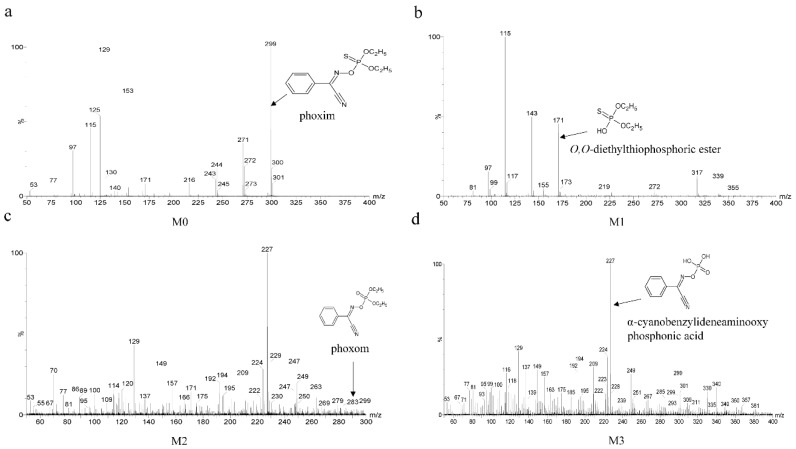
Mass spectra of the metabolic products of phoxim degradation by strain YP6. (**a**) Phoxim; (**b**) *O*,*O*-diethylthiophosphoric ester; (**c**) phoxom; (**d**) α-cyanobenzylideneaminooxy phosphonic acid.

The degradation intermediates of phoxim were characterized on an HPLC-MS system. In the HPLC spectrum (Figure 2), the retention time (RT) of phoxim (marked as M0) was 6.78 min, and its mass spectrum (Figure 3a) indicated a molecular ion at *m*/*z* 299 [M + H]^+^. The RTs of the other three peaks (marked as M1, M2, and M3) were 4.82 min, 4.48 min, and 6.15 min, respectively.

M1 was identified according to its mass spectrum characteristics. As shown in Figure 3b, its molecular ion was at *m*/*z* 171 ([M + H]^+^). As the ion is the protonated ion, the MW of M1 may be 170. An intermediate of phoxim hydrolyzation with MW of 170 has been previously identified as *O, O*-diethylthiophosphoric ester [1]. Furthermore, the characteristic ions at *m*/*z* 143 and 115 were formed by the loss of -C_2_H_5_ and -2C_2_H_5_, respectively. Thus, M1 was identified as *O, O*-diethylthiophosphoric ester, a hydrolysate formed by cleaving the P-aminooxy bond of phoxim. The result was consistent with our previous study, in which we found that an alkaline phosphatase (AP3) from strain YP6 could transform OPs (including phoxim) into corresponding hydrolytic products by cleaving the P-aminooxy bond [23]. The protonated ion of M2 was at *m*/*z* 283. Therefore, the MW of M2 was 282 and it was 16 Da less than that of phoxim, indicating that M2 might be produced by the desulfurization of phoxim to yield phoxom, which has been previously reported by Liu et al. [29]. Moreover, the fragment ion of M2 at *m*/*z* 227 was formed by loss of -2C_2_H_5_ from the protonated ion at *m*/*z* 283, and the fragment ion at *m*/*z* 149 was formed by the loss of -2C_2_H_5_ and -C_6_H_6_, and the characteristic fragment ion at *m*/*z* 129 was formed by cleaving the N–O bond (Figure 3c). For M3, the protonated ion at *m*/*z* 227 and the fragment ions at *m*/*z* 149 and 129 all correspond to characterized degradation products of phoxom (M2), and collectively indicate that M3 was α-cyanobenzylideneaminooxy phosphonic acid (Figure 3d).

### 2.4. Transcriptome Analysis

By comparing the RNA-seq data of strain YP6 cultivated with and without phoxim, a total of 1540 genes exhibited significant mRNA level changes, including 689 upregulated and 851 downregulated genes. In detail, a total of 176 differentially expressed genes (DEGs) were mapped to 26 gene ontology (GO) terms in the three main categories (molecular function, biological process, and cell component). DEGs that were upregulated in response to phoxim were found to be associated with various metabolic and regulatory processes, and most of these genes were affiliated to categories such as “catalytic activity”, “metabolic process”, “binding”, “cellular process”, and “cell part” (Figure S2). 

A total of 367 DEGs were classified into 25 major cellular processes based on Kyoto encyclopedia of genes and genomes (KEGG) annotation (Figure S3). All the DEGs involved in “cell motility”, including “bacterial chemotaxis” and “flagellar assembly”, were upregulated (Figure S3, Table S5). Cheng et al. [30] observed that during the transcriptomic analysis of *Rhodococcus erythropolis* D310-1 in response to chlorimuron-ethyl, five of the 15 unigenes involved in “Bacterial chemotaxis” were upregulated DEGs, and through the expression of these related genes, microorganisms can respond to contaminated environments and move towards contaminants, providing contaminant-degrading bacteria a stronger adaptive and contaminant-degrading capacity. Similarly, it is possible that the upregulated genes identified in this study as being involved in “bacterial chemotaxis” and “flagellar assembly” may play a role in the promotion of phoxim degradation of strain YP6.

Based on the metabolites analysis above, strain YP6 could transform phoxim into *O*,*O*-diethylthiophosphoric ester, phoxom, and α-cyanobenzylideneaminooxy phosphonic acid, via a series of oxidation, hydrolysis, and O-dealkylation enzyme-catalysed reactions. Analysis of transcriptomic data to identify which genes are upregulated by exposure to 50 mg L^−1^ of phoxim for 24 h enabled putative OP degradation genes to be identified (Table 3). This confirmed that transcription of the genes for various hydrolases was significantly enhanced, which corresponded with previously reported hydrolytic degradation of OPs [31,32,33,34,35]. Most significantly, the upgraded genes included two different alkaline phosphatases, which corresponded with the known ability of AP3 from YP6 to cleave the P-aminooxy bond of phoxim [23]. Therefore, our results indicated that strain YP6 over-expressed hydrolase activity encoding genes to improve phoxim hydrolysis. Various oxidative activities (aldehyde dehydrogenase, a number of monooxygenases, and NADPH-cytochrome P450 reductase) were also found to be significantly upregulated. They were reported to act on the desulfurization of the P=S group [36,37,38]. Notably, NADPH-cytochrome P450 reductase is a subunit of cytochrome P450 monooxygenase [39], an enzyme which is considered as one of most versatile biocatalysts [40], with confirmed O-dealkylation catalytic activity [41,42], indicating that it is an important gene in the degradation of phoxim by participating in the process of desulfurization and/or O-dealkylation. The transcriptome of strain YP6, in response to phoxim, also indicated that some upregulated genes identified as glycosyltransferase, which has been reported to be an important pesticide-detoxifying enzyme and to play important roles in the metabolism of pesticides [30,43].

### 2.5. qRT-PCR Validation

**Figure 4:**
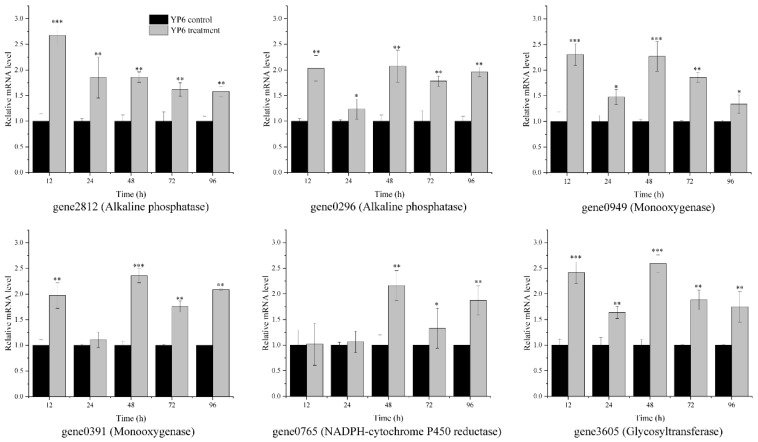
Differentially expressed genes (DEGs) involved in phoxim determined by qRT-PCR. Black and gray bars represent expression in samples without and with phoxim, respectively. The absolute expression levels are shown with the 16S rRNA used as an endogenous control. Statistically significant differences are marked with * (*p*-value < 0.05), ** (*p*-value < 0.01), or *** (*p*-value < 0.001) using Student’s *t*-Test.

To verify our preliminary predicted genes involved in phoxim degradation of strain YP6, we selected the genes encoding four enzymes and examined the changes in their transcription levels by using qRT-PCR at different culture times. Compared to the control group, the mRNA expressions of the six genes were almost all significantly upregulated over 96 h (Figure 4). The six genes were encoding alkaline phosphatases (gene2812 and gene 0296), monooxygenases (gene 0949 and gene0391), NADPH-cytochrome P450 reductase (gene0765), and glycosyltransferase (gene3605), respectively. The overexpression of these genes (except for gene3605), and the activity of the corresponding enzyme also increased, enabling strain YP6 to transform phoxim into *O*,*O*-diethylthiophosphoric ester, phoxom, and α-cyanobenzylideneaminooxy phosphonic acid. Glycosyltransferase is an important pesiticide-detoxifying enzyme and can combine hydrolysates of pesticides with glucuronic acid, glucose, sulfuric acid, or glutathione, thereby increasing the solubility of pesticides and decreasing their toxicity. Moreover, gene3605, identified as a glycosyltransferase, was also overexpressed during 96 h exposure to phoxim, indicating that glycosyltransferase plays a pivotal role in the process of phoxim degradation by strain YP6. This may be the reason why α-hydroxyiminophenylacetonitrile, the other hydrolysate of phoxim, was not detected using both HPLC and HPLC-MS methods.

### 2.6. Biodegradation Pathway of Phoxim by Strain YP6

**Figure 5:**
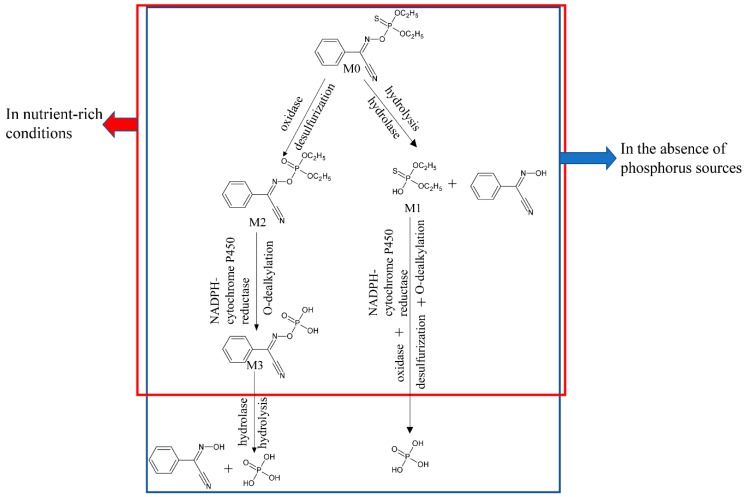
Proposed metabolic pathway of phoxim by strain YP6.

In nutrient-rich conditions (LB broth medium), the results of HPLC-MS showed that strain YP6 transformed phoxim into *O*,*O*-diethylthiophosphoric ester, phoxom, and α-cyanobenzylideneaminooxy phosphonic acid. By analyzing the transcription data of strain YP6 cultivated in LB with and without phoxim, it was observed that the genes corresponding to various hydrolytic (phosphatases, hydrolases, esterase) and oxidative (aldehyde dehydrogenase, monooxygenases, NADPH-cytochrome P450 reductase) enzymes were upregulated significantly. Based on these outcomes, the pathways for phoxim degradation by YP6 were proposed and are presented in Figure 5. The degradation of the OP pesticide by this bacterium involved two different metabolic routes. In the hydrolytic pathway, phoxim was initially cleaved to *O*,*O*-diethylthiophosphoric ester, whereas in the oxidative pathway, phoxim was initially transformed into phoxom by desulfurization, and subsequently into α-cyanobenzylideneaminooxy phosphonic acid by O-dealkylation. A comparison of the relative peak areas of the three metabolites generated after 48 h of phoxim degradation by YP6 indicated that *O*,*O*-diethylthiophosphoric ester production was significantly greater than that of the other two characterized metabolites. Therefore, the hydrolytic pathway is presumed to be the main one used by YP6. Additionally, YP6 was found to utilize phoxim as a sole phosphorus source. Therefore, in the absence of other available phosphorus sources, the intermediates of the two pathways would be further degraded into phosphoric acid by successive O-dealkylation and then either hydrolysis or desulfurization, thereby supplying the phosphorus necessary for growth. These data showed that strain YP6 could degrade phoxim both in nutrient-rich conditions and in the absence of phosphorus sources. To date, most studies on phoxim have focused on its effect on animals, especially *Bombyx mori* [30,44,45,46], and only a relative few reports have focused on the biodegradation of phoxim. To our best knowledge, this study presents a previously unreported microbial phoxim degradation pathway. However, the key genes involved in phoxim-degradation still require further study and application in OP degradation.

## 3. Materials and Methods

### 3.1. Pesticides, Strain and Cultivation Medium

Technical phoxim (99.7%) was purchased from Sigma–Aldrich (Shanghai, China) and used as the standard for HPLC and HPLC-MS detections. Chemical phoxim (40%) was purchased from a local market and used for phoxim degradation experiments. Other chemical reagents, unless otherwise specified, were all analytical grade.

Strain *B. amyloliquefaciens* YP6 was isolated from a phosphate mine in Guizhou Province and deposited in the China Center for Type Culture Collection (deposit ID: CCTCC NO: M 2018875).

Luria–Bertani broth (LB broth) was prepared for the cultivation of the strain.

Three different mineral salt media (MSM) and their components are shown in Tables S1–S3. After adding a final phoxim concentration of 50 mg L^−1^, the three MSM media were named M-1 (phoxim as a sole carbon source), M-2 (phoxim as a sole phosphorus source), and M-3 (phoxim as a sole carbon−phosphorus source), respectively. Solid medium was made with a 2.0% agar supplement.

### 3.2. The Growth and Phoxim Degradation of Strain YP6 in Different Culture Media

Strain YP6 was cultured in LB broth at 30 °C and 200 rpm until reaching the exponential phase. The culture medium was centrifuged (8000× *g*, 5 min, 4 °C). The harvested cells were washed three times with sterile distilled water and suspended in the sterile distilled water to approximately 0.6 of OD_600_ by UV-3900 (HITACHI, Tokyo, Japan). Each 100 μL of cell suspension was spread on LB broth (containing 50 mg L^−1^ of phoxim), M-1, M-2, and M-3 agar plates, respectively. These agar plates were incubated at 30 °C for 7 days. Following the steps above, cell suspension was inoculated (3% of inoculation, *v*/*v*) into the liquid medium, which was chosen according to the growth status of YP6 on different agar plates. Each experiment was repeated three times. Control groups either contained phoxim but were not inoculated with bacterial cells or were inoculated with bacterial cells but without the addition of phoxim. Finally, the medium was incubated at 30 °C, 200 rpm. Samples were taken at day 0, 0.5, 1, 2, 3, 4, 5, 6, and 7 for further analysis.

### 3.3. Response Surface Experimental Design for Phoxim Degradation

The preliminary degradation screening showed that strain YP6 could rapidly degrade phoxim in an LB broth medium. RSM was used to optimize the parameters that significantly affected biodegradation by strain YP6. Design-Expert 7.1.3 software (Stat-Ease Inc., Minneapolis, MN, USA) was used for the experimental design and data analysis. Three variables: initial inoculum (1%, 3%, 5%, *v*/*v*), incubation temperature (20, 30, 40 °C) and pH (5.0, 7.0, 9.0) were considered as important factors for phoxim degradation [27]. Thus, they were chosen as the independent variables and designated as factors A (incubation temperature), B (pH), and C (initial inoculum), respectively. Each factor was varied over 3 levels. The degradation studies were performed in 250 mL Erlenmeyer flasks containing sterile LB broth, bacterial suspension and phoxim (50 mg L^−1^ of final concentration), which was incubated on a rotary shaker at 200 rpm. The samples were tested at 48 h. Each sample was repeated three times. Control groups either contained phoxim but were not inoculated with bacterial cells or were inoculated with bacterial cells but without the addition of phoxim.

### 3.4. HPLC Analytical Methods

The pretreatment method of phoxim was referred to in Deng et al. [15], and the specific operation was as follows: phoxim samples (500 μL) were added to equal volumes of methanol, then shaken vigorously and centrifugated at 12,000× *g* for 10 min; Finally, the supernatants were passed through a 0.22-μm filter. They were then quantified by HPLC. A Hitachi chromaster system equipped with an Agilent ZORBAX SB-C18 (5 μm, 4.6 × 250 mm) was used. The detection conditions of phoxim were referred to in Deng et al. [15].

### 3.5. Identification of Metabolites of Phoxim by HPLC-MS

Strain YP6 was cultured in LB broth medium with phoxim (an initial concentration of 50 mg L^−1^) under the optimum biodegradation conditions for 48 h. LB broth with phoxim was used as a control. Samples were extracted according to Lin et al. [1]. The extracts containing phoxim were analyzed by HPLC-MS. The system, column, and method were the same as those used in Li et al. [47]. The different conditions were as follows: mobile phase A (methanol), mobile phase B (H_2_O, 0.1% formic acid), 0.3 mL/min of flow rate, 1 µL of sample size, a source temperature of 100 °C, a desolvation temperature of 400 °C, capillary voltage of 3.5 kV, and cone voltage of 30 V.

### 3.6. RNA-seq Transcriptomic Analysis

A single-strain YP6 colony on an M-2 agar plate was inoculated in LB broth medium at 30 °C and 200 rpm until reaching the exponential phase. The culture medium was centrifuged (8000× *g*, 5 min, 4 °C). The harvested cells were washed three times with sterile distilled water and suspended in the sterile distilled water to approximately 0.6 of OD_600_. Then, the cell suspension was inoculated (1% of inoculation, *v*/*v*) in LB broth medium with or without 50 mg L^−1^ phoxim at 30 °C, 200 rpm for 24 h. Triplicate cultures with added phoxim (an initial concentration of 50 mg L^−1^) were treated samples (named YP6 treatment). In parallel, the other three cultures without phoxim were control groups (named YP6 control). All samples were centrifugated at 4 °C, 8000× *g* for 10 min. Each sample was treated with TRIzol Reagent (Invitrogen, Carlsbad, CA, USA)/RNeasy Mini Kit (Qiagen, Hilden, Germany) and immediately frozen in liquid nitrogen and delivered to GENEWIZ for transcriptome sequencing analysis. In brief, total RNA was extracted from bacterial cells and then rRNA was removed. Purified mRNA was fragmented and used as templates for cDNA synthesis. The cDNA libraries were sequenced on an Illumina HiSeq instrument (Illumina, San Diego, CA, USA). The genome of strain YP6 (CP 032146) was used as a reference for alignment and annotation. A total of 22,391,690 reads mapped to the reference genome in the sample of the YP6 control, and 17,752,954 reads in the sample of YP6 treatment. DEGs were assigned by EdgeR (v3.4.6, Bioconductor, Buffalo, NY, USA) with an adjusted *p*-value < 0.05 [48]. GO terms that annotate DEGs were used by GO-TermFinder (v0.86, Lewis-Sigler Institute, Princeton, NJ, USA). The statistical enrichment of DEGs in KEGG pathways was performed using scripts inhouse.

### 3.7. qRT-PCR Analysis

To confirm the results of RNA-Seq and quantify the transcription of key genes related to phoxim degradation of strain YP6, a qRT-PCR using Ultra SYBR Mixture (CW0957S, CWBIO) was performed and the primers used were listed in Table S6. YP6 control (without phoxim) and treatment samples (containing an initial phoxim concentration of 50 mg L^−1^) were both cultured at 30 °C, 200 rpm. Samples were collected at 12, 24, 48, 72, and 96 h, respectively. Total RNA of each sample was extracted and purified as per the steps mentioned in the section “RNA-seq transcriptomic analysis”. Then, cDNA was synthesized from total RNA with random primers by using HiScript^®^ II Q RT SuperMix for qRT-PCR (R222-01, Vazyme, Nanjing, China). Finally, the obtained cDNA was used as a template for qRT-PCR. The 16s rRNA gene was chosen as an internal control. All reactions were performed in three biological replicates, and the 2^−△△CT^ method [30] was used to calculate the gene expression levels for strain YP6.

### 3.8. Statistical Analysis

For RNA-Seq data, the EdgeR Bioconductor package (v3.4.6, Bioconductor, Buffalo, NY, USA) was used for differential expression analysis. FDR, controlling for the false discovery rate, was used to adjust the *p*-values. Genes with a *p*-value < 0.05 were assigned as differentially expressed. For the qRT-PCR experiments, the data were analyzed by ANOVA and a *p*-value < 0.05 was considered to have statistical significance.

For degradation of phoxim, the data calculation was as follows:

$$\mathrm{Degradation}\;\left(\%\right)=\frac{\left(\mathrm{Residual}\;\mathrm{amount}\;\mathrm{in}\;\mathrm{blank}\;\mathrm{control}-\mathrm{residual}\;\mathrm{amount}\;\mathrm{in}\;\mathrm{sample}\right)}{\mathrm{Residual}\;\mathrm{amount}\;\mathrm{in}\;\mathrm{blank}\;\mathrm{control}}\times 100$$

For OD_600_ of strain YP6, the data calculation was as follows:

$${\mathrm{OD}}_{600}={\mathrm{OD}}_{600}\;\mathrm{at}\;\mathrm{different}\;\mathrm{cuture}\;\mathrm{time}-\;{\mathrm{OD}}_{600}\;\mathrm{at}\;0\;d$$

## 4. Conclusions

In this work, *B. amyloliquefaciens* YP6, possessing both plant growth-promoting ability [22] and the metabolic capacity to efficiently degrade a wide range of OPs [23,24], was used for studying phoxim degradation. It was shown that strain YP6 could grow and degrade phoxim efficiently in LB broth medium. It could also utilize phoxim as the sole phosphorus source in a mineral salt medium. This strain could degrade 96.12% of phoxim under optimum degradation conditions (40 °C, pH 7.20, and inoculum size of 4.17% (*v*/*v*)) in 48 h. Additionally, strain YP6 could transform phoxim into *O*,*O*-diethylthiophosphoric ester, phoxom, and α-cyanobenzylideneaminooxy phosphonic acid in the LB broth medium, while these intermediates were found to further degrade into phosphoric acid in an M-2 medium. The information provided here not only helps us to understand the phoxim degradation by strain YP6, but can also be used as a reference for phoxim degradation conditions in pesticide-contaminated sites.

## Figures and Tables

**Table 1:** Random experimental design and results based on response surface methodology (RSM) for phoxim degradation.

Run	A (Temperature, °C)	B (pH)	C (Initial Inoculum, *v*/*v*, %)	R1 (Degradation, %)
1	30	5	5	74.9 ± 0.5
2	40	7	5	95.3 ± 0.2
3	30	7	3	85.6 ± 1.1
4	30	9	1	76.0 ± 0.4
5	40	5	3	86.8 ± 0.9
6	30	7	3	85.1 ± 1.4
7	30	7	3	84.6 ± 0.6
8	30	5	1	73.0 ± 0.5
9	40	7	1	92.4 ± 0.3
10	30	7	3	85.5 ± 0.5
11	20	9	3	67.1 ± 0.2
12	20	7	1	73.7 ± 0.7
13	30	9	5	78.6 ± 0.8
14	30	7	3	85.6 ± 1.3
15	20	7	5	71.4 ± 0.4
16	40	9	3	89.4 ± 0.7
17	20	5	3	64.3 ± 0.9

**Table 2:** ANOVA results of quadratic model.

Source	Sum of Squares	Freedom	Mean Square	*F* Value	*p* Value	
Model	1273.56	9	141.51	285.83	<0.0001	significant
A	954.84	1	954.84	1928.70	<0.0001	
B	18.30	1	18.30	36.97	0.0005	
C	3.25	1	3.25	6.57	0.0374	
AB	0.01	1	0.01	0.02	0.8910	
AC	6.76	1	6.76	13.65	0.0077	
BC	0.12	1	0.12	0.25	0.6341	
A2	0.68	1	0.68	1.38	0.2789	
B2	267.96	1	267.96	541.26	<0.0001	
C2	11.85	1	11.85	23.93	0.0018	
Residual	3.47	7	0.50			
Lack of Fit	2.72	3	0.91	4.84	0.0808	not significant
Pure error	0.75	4	0.19			
Cor Total	1277.02	16				

Note: R^2^ = 0.9973; Adj R^2^ = 0.9938.

**Table 3:** Upregulated genes related to phoxim biodegradation in *B. amyloliquefaciens* YP6.

Annotation	Gene ID	Gene Length (bp)	Log2 Ratio	*p*-Value	False Discovery Rate (FDR)	KEGG	GO
Alkaline phosphatase	gene2812	1704	3.3402	8.19 × 10^−6^	9.52 × 10^−5^	-	Catalytic activity
Alkaline phosphatase	gene0296	1752	2.3702	0.001386	0.006986	Aminobenzoate degradation…	Catalytic activity, cellular process, metabolic process
Hydrolase	gene3280	822	2.5297	0.001637	0.007887	-	Metabolic process, hydrolase activity
Hydrolase	gene3724	681	2.6083	0.002753	0.011817	-	Catalytic activity, membrane, membrane part, cellular process, metabolic process, single-organism process
Hydrolase	gene0396	1152	2.0757	0.004794	0.01811	-	Catalytic activity, metabolic process
Esterase	gene1642	780	2.0589	0.017248	0.048692	-	Catalytic activity, cellular process, metabolic process, single-organism process
Aldehyde dehydrogenase	gene4167	1338	1.7844	0.017696	0.049755	Chloroalkane and chloroalkene degradation…,	
Monooxygenase	gene0949	1137	2.5757	0.000289	0.002002	…	Catalytic activity, metabolic process, single-organism process
Monooxygenase	gene0391	1323	2.3294	0.002035	0.009346	-	Catalytic activity, metabolic process, single-organism process
Monooxygenase	gene3517	1329	2.0024	0.005881	0.021023	-	Catalytic activity, metabolic process, single-organism process
NADPH-cytochrome P450 reductase	gene0765	3186	1.6887	0.014603	0.042848	Aminobenzoate degradation…	Catalytic activity, binding, metabolic process, single-organism process
Glycosyltransferase	gene3605	843	3.5212	1.86 × 10^−5^	0.000197	-	Catalytic activity
Glycosyltransferase	gene3602	1038	3.6099	0.000124	0.000985	-	Catalytic activity
Glycosyltransferase	gene3604	1137	2.9008	0.000162	0.001233	-	Catalytic activity
Glycosyltransferase	gene1749	1704	2.6475	0.000421	0.002651	-	Catalytic activity
Glycosyltransferase	gene3606	1140	2.3595	0.001376	0.006966	-	Catalytic activity
Glycosyltransferase	gene3242	846	2.3983	0.002447	0.010743	-	Catalytic activity
Glycosyltransferase	gene0462	1263	1.8593	0.012085	0.037201	-	Catalytic activity, membrane, membrane part
Glycosyltransferase	gene3982	807	2.1159	0.012498	0.038173	-	Catalytic activity
Glycosyltransferase	gene3600	1035	2.1363	0.012623	0.038501	-	Catalytic activity

“…” contain other pathway; “-” Not matched to KEGG.

## Supplementary Materials

The following are available online, Figure S1: The growth curve of strain YP6 in LB broth medium (containing an initial concentration of 50 mg L^−1^ phoxim) at different temperature, Figure S2: Expression profiles of DEGs based on GO annotation, Figure S3: Expression profiles of DEGs based on KEGG annotation, Table S1: M-1 (lacking carbon source), Table S2: M-2 (lacking phosphorus source), Table S3: M-3 (lacking carbon and phosphorus source), Table S4: Growth status of strain YP6 on different solid media, Table S5: Upregulated genes involved in cell motility in *B. amyloliquefaciens* YP6, Table S6: qRT-PCR primers used in this study.
