# Supplementary material for: Evaluation of the Strain Bacillus amyloliquefaciens YP6 in Phoxim Degradation via Transcriptomic Data and Product Analysis
Source: Molecules. 2019 Nov 5;24(21):3997. doi: 10.3390/molecules24213997 (PMC6864786; doi:10.3390/molecules24213997)
Supplement: Supplementary file 1 [file molecules-24-03997-s001.pdf]

Supporting information

Table S1. M-1 (lacking carbon source)

Component	Concentration (g/L)
$(NH_4)_2SO_4$	1.0
KH_2PO_4	0.5
K_2HPO_4	1.5
$MgSO_4 \cdot 7H_2O$	0.1
NaCl	1.0

Table S2. M-2 (lacking phosphorus source)

Component	Concentration (g/L)
$(NH_4)_2SO_4$	1.0
glucose	10
KCl	1.0
$MgSO_4 \cdot 7H_2O$	0.1
NaCl	1.0

Table S3. M-3 (lacking carbon and phosphorus source)

Component	Concentration (g/L)
$(NH_4)_2SO_4$	1.0
KCl	1.0
$MgSO_4 \cdot 7H_2O$	0.1
NaCl	1.0

Table S4. Growth status of strain YP6 on different solid medium

Solid medium	LB broth [a]	M-1	M-2	M-3
Growth status	+	-	+	-

[a] contained a final phoxim concentration of 50 mg/L; "+" Strain YP6 grew on this solid medium; "-" strain YP6 could not grow on the solid medium.

Table S5. Upregulated genes involved in cell motility in *B. amyloliquefaciens* YP6.

Annotation	Gene ID	Gene name	Gene length (bp)	log2 Ratio	P-value	FDR
**Cell motility**						
**Flagellar assembly**						
Flagellar assembly protein FliH	gene1765	*fliH*	756	4.4407	9.48E-06	0.000109
Flagellar motor protein MotS	gene3106	*motB*	621	4.7310	4.34E-05	0.000404
Flagellar basal body rod modification proteinFlgD	gene1770	*flgD*	432	9.4147	7.38E-05	0.000635
Flagellar motor protein MotP	gene3107	*motA*	816	2.7941	0.000273	0.001910

Flagellar motor protein MotA	gene1475	*motA*	816	3.6705	0.000456	0.002846
ATP synthase	gene1766	*fliI*	1317	2.5511	0.000802	0.004436
Flagellar motor switch protein FliG	gene1764	*fliG*	1017	2.3578	0.002157	0.009743
Flagellar hook-length control protein	gene1769	*fliK*	1344	2.2450	0.002455	0.010760
Flagellar biosynthesis protein FlhA	gene1782	*flhA*	2034	2.1108	0.002885	0.012222
Flagellar basal body rod protein subunit C	gene3814	*flgG*	798	2.2864	0.002985	0.012551
RNA polymerase sigma factor SigD	gene1790	*fliA*	765	1.9124	0.007816	0.026351
Flagellar basal-body M-ring protein	gene1763	*fliF*	1533	1.8968	0.008985	0.029553
Flagellar protein FliT	gene3701	*fliT*	345	7.9145	0.009775	0.031551
Flagellar motor protein MotB	gene1474	*motB*	804	1.9640	0.014806	0.043267
**Bacterial chemotaxis**						
Chemotaxis protein	gene3254	*mcp*	1989	2.9952	4.68E-05	0.000432
Chemotaxis response regulator protein-glutamatemethylesterase	gene1785	*cheB*	1068	3.2791	0.000121	0.000967
Methyl-accepting chemotaxis protein	gene1504	*mcp*	2079	2.7564	0.000191	0.001419
Chemotaxis protein	gene3252	*mcp*	2007	2.6552	0.000323	0.002178
Chemotaxis protein	gene3253	*mcp*	1986	2.6444	0.000398	0.002529
Chemotaxis protein CheD	gene1789	*cheD*	501	2.6934	0.003539	0.014337
Methyl-accepting chemotaxis proteins (MCPs)methyltransferase	gene2430	*cheR*	933	2.3897	0.004213	0.016494
Chemotaxis protein	gene3255	*mcp*	1986	1.9330	0.006375	0.022383
Chemotaxis protein CheA	gene1786	*cheA*	2019	1.9346	0.007067	0.024421
Response regulator	gene1940	*cheY*	363	2.0412	0.013548	0.040481

"-" Not located to the gene name.

Table S6. qRT-PCR primers used in this study

**Name**	**Sequence (5' to 3')**
16s rRNA-F	GAAGTCGTAACAAGGTAG
16s rRNA-R	CAAGGTCTTATATTCCGTTA
gene0296-F	GGACCGAATGAATATGTG
gene0296-R	GGTAATCTGCCAGTGTAT

gene2812-F	AAGTGTAGAGCAGGAGAAT
gene2812-R	CGAGAGTGTGATTGATACG
gene0765-F	CGTCTTGATGTTCAGGATA
gene0765-R	TCTGCGATAATACTGTCTAC
gene3605-F	GACATTGGCGGAGAGTATT
gene3605-R	GCGTCATCACACATAATCAG
gene0949-F	TTGCTCGTAAATGTCGTA
gene0949-R	ATTCTTCAGTCGCCTTAT
gene0391-F	AAGCATCTTGAGCACCAT
gene0391-R	CGAATCCCATAATCCTTTGAC

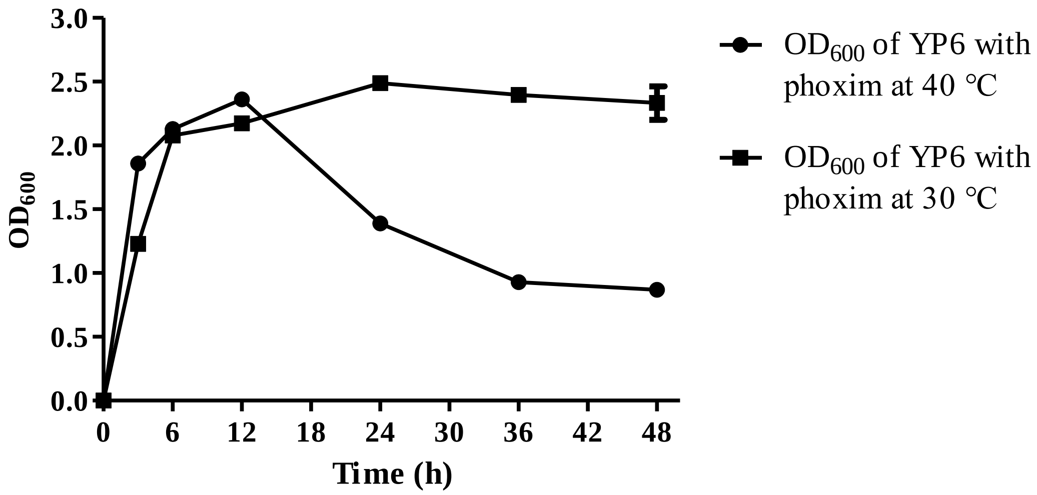

Figure S1. The growth curve of strain YP6 in LB broth medium (containing an initial concentration of 50 mg L^{-1} phoxim) at different temperature. The specific operation was as follows: firstly, strain YP6 was cultured in LB broth at 30 ℃ and 200 rpm until reaching the exponential phase; then, the cell suspension was inoculated (4.17% of inoculation, v/v) in LB broth medium (containing an initial concentration of 50 mg L^{-1} phoxim) at 30 ℃ and 40 ℃, 200 rpm. For OD_{600} of YP6, the data calculation was as follows: $OD_{600} = OD_{600}$at different culture time $-$ OD_{600} of initial inoculation.

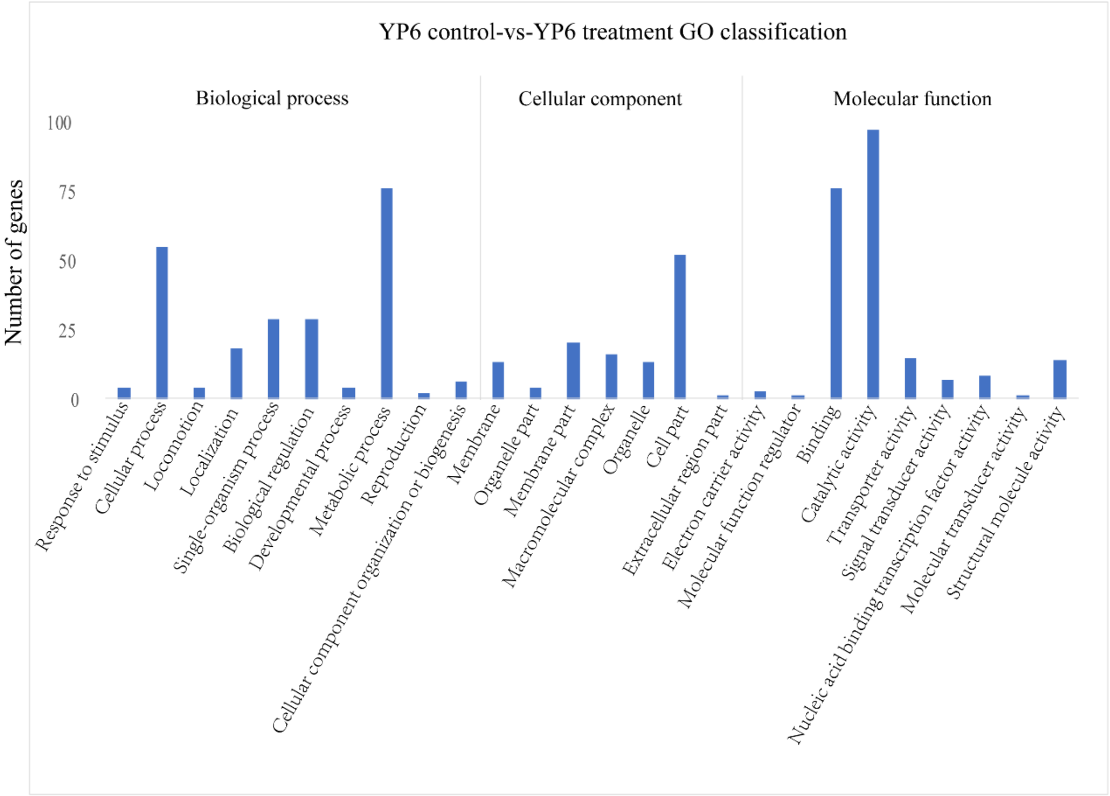

Figure S2. Expression profiles of DEGs based on GO annotation.

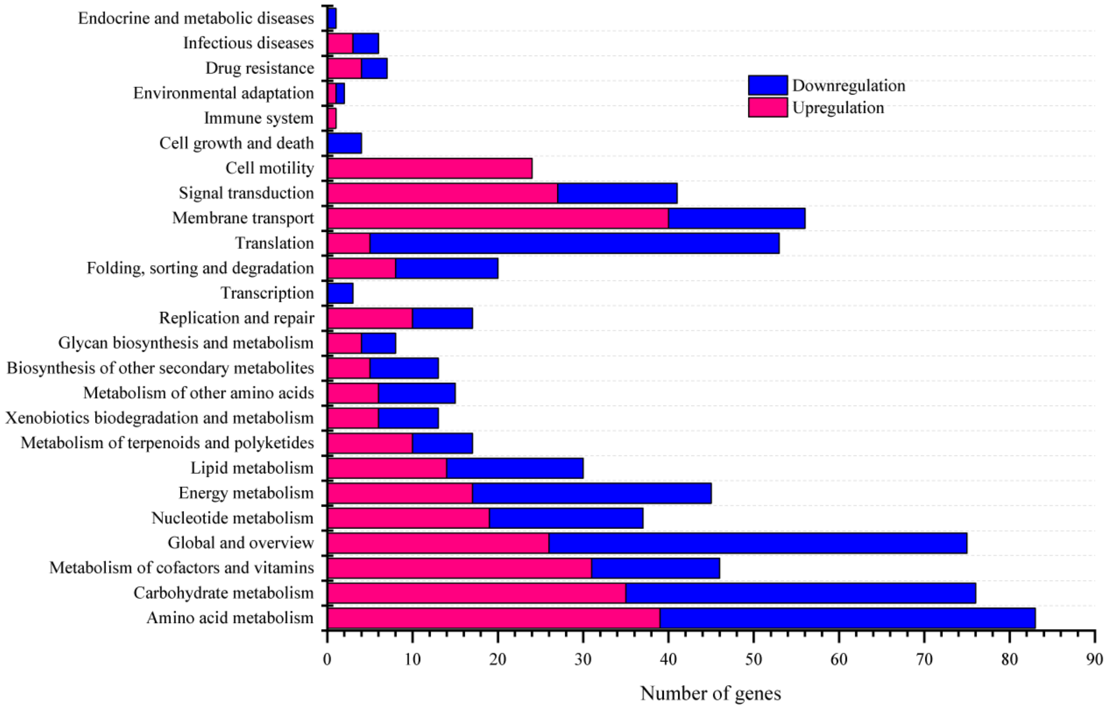

Figure S3. Expression profiles of DEGs based on KEGG annotation.
